# A Pilot Study on the Influence of Self-Paced Auditory Cues and Preferred Music on Gait in Persons with Parkinson’s Disease

**DOI:** 10.3390/brainsci15050528

**Published:** 2025-05-20

**Authors:** Maddie Brant, Callan Barrick, Lindsay Muno, Elizabeth Stegemoller

**Affiliations:** 1 Department of Neuroscience, Iowa State University, Ames, IA 50011, USA; maddie.e.brant@gmail.com; 2 Department of Kinesiology, Iowa State University, Ames, IA 50011, USA; callanbarrick@gmail.com (C.B.); lindsayamuno@gmail.com (L.M.)

**Keywords:** Parkinson’s disease, auditory cueing, gait disturbance, gait stability, preferred walking speed, therapeutic intervention, music-cued gait, metronome-cued gait

## Abstract

**Background**: Gait disturbance in Parkinson’s Disease (PD) significantly impacts quality of life and is not completely mitigated by dopaminergic treatment. Auditory cueing has been shown to help improve certain aspects of gait, but its effects when matched to individuals’ preferred walking rate remain unexplored. **Methods**: Nine individuals with PD walked at their preferred rate across a GAITRite^®^ mat under three separate conditions: self-paced, metronome-cued, and music-cued. Spatiotemporal gait measures were collected and analyzed using repeated measures ANOVAs and post-hoc paired-samples *t*-tests. **Results**: A main effect of condition was revealed for step width (F = 3.533, *p* = 0.054, η_p_^2^ = 0.306), with reduced step width revealed during the music-cued condition. Post-hoc analysis revealed no significance (*p* > 0.063). **Conclusions**: The trend in step width data suggests a potential benefit of music cueing for enhancing gait stability in persons with PD. Results of this pilot study provide valuable framework for future research and the development of therapeutic interventions to enhance gait stability, reduce fall risk, and improve overall quality of life.

## 1. Introduction

One of the greatest struggles that people with Parkinson’s disease (PD) face is gait disturbance, which becomes progressively worse over time [1]. Common characteristics of Parkinsonian gait include stooped posture, freezing, festination, shuffling, and falling [2]. Current treatment strategies for PD are not always effective in ameliorating the symptoms of Parkinsonian gait. For example, although dopaminergic medication has been shown to enhance balance and improve performance of gait tasks, significant impairments still exist when comparing individuals with PD to healthy adults [3]. Moreover, dopaminergic medications do not affect both spatial and temporal gait parameters equally; gait characteristics, like speed and stride length, may be improved by medication, whereas temporal parameters such as cadence, step duration, swing duration, and double support time can be completely unaffected by dopaminergic treatments [4]. When left untreated, gait impairments can lead to falls, so it is essential to find alternative strategies to supplement medication and reduce risks of injury among individuals with PD.

Aside from medicinal intervention (i.e., L-dopa), another primary strategy for treating Parkinsonian gait is external cueing. Several different types of external cueing (i.e., visual, auditory, verbal instructions, and somatosensory cueing) have been used in research studies to assess their efficacy in being able to influence spatiotemporal characteristics of gait [5]. One type of cueing, visual cueing, involves the placement of visual markers on the floor as guidelines for participants to follow whilst they are completing a gait task. A review conducted by Muthukrishnan et al. [6] highlighted multiple studies that demonstrate how visual cueing can lead to benefits like increased gait speed and step length, therefore supporting its efficacy as a treatment strategy for gait disturbances. Auditory cueing is another common type of cueing used in PD research studies and has been shown to influence gait by recruiting existing connections between the auditory and motor systems and synchronizing the precise timing mechanisms of body movement and rhythm to the timing of particular patterns of sound [7,8]. Metronomes and music are two specific forms of auditory cueing that facilitate gait improvements via the phenomena of entrainment, synchronization, and pace stabilization [8]. Several studies have uncovered the effects of metronome cueing and music on PD gait by altering participants’ walking speeds [9,10,11]. Ready et al. [12], for example, observed that rhythmic auditory stimulation was linked to regulation of cadence and increased gait velocity of both young and older adults. However, the effects of metronome cues and music on gait parameters have yet to be investigated when the participants’ experimental walking speeds are matched with their preferred walking rates in persons with PD.

Therefore, the purpose of this pilot study was to investigate the effects of metronome cues and music (presented separately) on spatiotemporal measures of gait when cued at individuals’ preferred walking rate. We hypothesize that spatiotemporal gait parameters will improve when walking with both a metronome and music cues at the preferred walking rate of participants with PD.

## 2. Materials and Methods

### 2.1. Participants

A convenience sample of 9 individuals with PD participated in this pilot study, 7 of which were female and 2 were male, with an average age of 68 ± 4.4 years. All participants were of white ethnicity and the average PD duration was 7.4 ± 5.0 years. All participants were tested on medication and maintained a stable regimen of antiparkinsonian medications for 30 days prior to participation in the study. Participants displayed no clinical symptoms of cognitive impairment (Mini-Mental State Exam, MMSE = 29.7 ± 0.7) or major depression (Beck Depression Inventory, BDI = 5.7 ± 3.1). In addition, all participants were able to ambulate independent of an assistive device in order to accurately measure changes in gait across different conditions. Informed consent, approved by the institutional review board of Iowa State University, was obtained from all participants prior to completing additional pre-participation screening procedures.

After consent was obtained, the Movement Disorder Society—Unified Parkinson’s Disease Rating Scale (MDS-UPDRS) was completed for each participant. Table 1 shows participant demographic and disease information.

### 2.2. Data Collection

When comfortable, participants were asked to walk 10 m, at their preferred rate, across an electronic walkway (GAITRite^®^) designed to detect foot pressure. When using cues for walking in a clinical environment, the cue (metronome or music) rate is typically matched to the patient’s self-selected pace. Thus, the preferred rate was chosen to reflect clinical practice. Participants completed 5–7 trials and the preferred walking speed was calculated before completing the next two cued walking conditions. Using the preferred walking rate, participants then completed 5–7 trials of walking with a metronome cue set at the preferred rate. Participants were then asked to provide the name and artist of their most preferred song. The tempo of this selected song was then adjusted using Pitch Switch version 4.0.3 (Inspyder Software Inc., 2011, Burlington, ON, Canada) to ensure it matched the rate of preferred walking without major distortion. If the adjusted tempo of the song appeared to be too distorted, participants were asked to provide a second song choice to better match their preferred walking rate. Participants then completed 5–7 trials of walking with their selected music as the preferred rate.

### 2.3. Data Analysis

GAITRite software version 4.8 was used to obtain measures of spatiotemporal gait parameters of cadence, stride length, step width, step length, step time, stance time, swing time, single support time, and double support time. Data were inspected for outliers and then averaged under each walking condition. To address the stated hypothesis, a one-way repeated measures analysis of variation (ANOVA) was completed to compare spatiotemporal gait measures between the three walking conditions. Post-hoc analyses were completed using a paired-samples *t*-test adjusted with Bonferroni correction. Effect size was calculated using partial eta squared. Significance was set at *p* < 0.05.

## 3. Results

**Figure 1:**
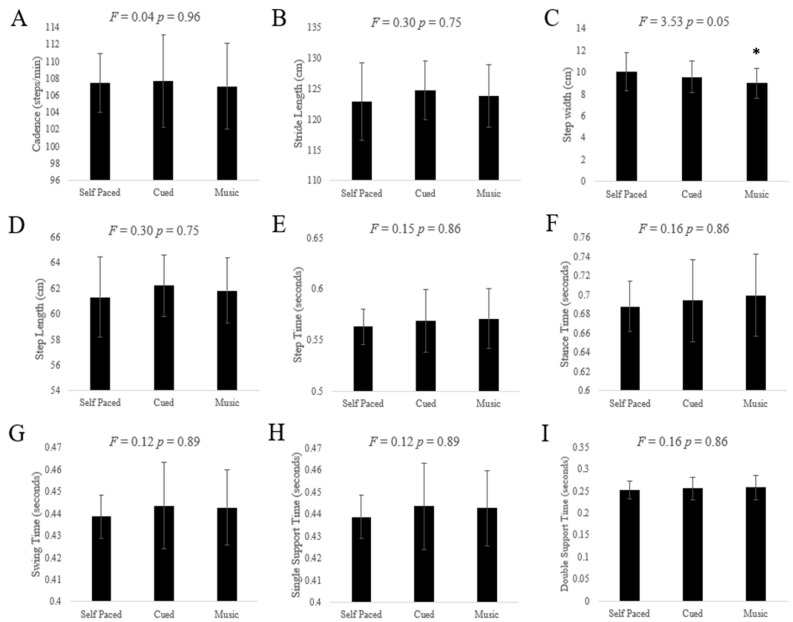
(**A**–**I**) Graphs of spatiotemporal gait measures by condition. * = statistically significant difference in music-cued step width data.

All three conditions, self-paced, cued, and music, produced similar values for each spatiotemporal gait measure, except for step width. A main effect of condition was revealed for step width (F = 3.533, *p* = 0.054, η_p_^2^ = 0.306); step width was revealed to be smaller during the music-cued condition compared to the self-paced and metronome-cued conditions. Given the large effect size, exploratory post-hoc analyses were conducted to further examine group differences and inform future research. However, post-hoc analyses revealed no significant differences between conditions (*p* > 0.063). There was no main effect for any condition across the remaining eight spatiotemporal measures. Trends shown on individual graphs of spatiotemporal measures (Figure 1) are not supported by statistical significance.

## 4. Discussion

This pilot study was designed to investigate the effects of metronomic and musical cues on Parkinsonian gait when cues are matched to participants’ preferred walking rate. We hypothesized that spatiotemporal measures of Parkinsonian gait would improve from non-cued to both metronome- and music-cued walking conditions. The goal of this study was to better inform the development of treatment strategies for managing walking difficulties in persons with PD, ultimately enhancing functional mobility and overall quality of life.

After visual inspection of graphical outputs for each spatiotemporal measure, few differences in spatiotemporal gait measures between each of the three experimental conditions were revealed. Of the nine total metrics, a main effect of condition was found for only step width, where step width was shown to be smaller under the music-cued condition compared to self-paced and metronome-cued conditions. However, post-hoc analysis did not reveal statistical significance in the difference between step width values for each condition.

The main effect of condition on step width could be the result of variations in factors such as motor control mechanisms, attentional focus, or movement timing. Morris et al. [13] hypothesized a neural mechanism for external cueing (i.e., auditory cueing), which favors the dorsolateral pre-motor control system, as opposed to the supplementary motor area (SMA). The SMA, together with the basal ganglia, plays a role in the body’s internal timing network [14]; therefore, dysfunction in this region, as seen in PD, negatively impacts temporal gait measures. Bypassing the dysfunctional SMA could help improve certain aspects of Parkinsonian gait [15]. Temporal gait measures like cadence and gait speed have been shown to improve with external auditory cueing, and this altered motor control mechanism could provide an explanation for why a spatial gait measure like step width varied between conditions in this study [6].

Alternatively, changes in step width could be explained by the influence of external cues on attentional focus. Auditory cues can be responsible for shifting attention towards different stimuli, i.e., aspects of the gait cycle or the surrounding environment, and this can have both positive and negative effects on gait [16]. For example, auditory cues may help people with PD synchronize their movements to an external beat, compensating for impaired internal timing [17]. However, these cues can also be distracting, requiring additional attentional resources, and potentially reducing gait quality and increasing the risk of falls [18]. In this study, participants may have adjusted their normal gait pattern to match an external auditory cue, a process known as sensorimotor synchronization [19], which could explain the changes in step width. Synchronization not only involves attentional shifts but also affects gait timing and coordination [20]. Changes in step width could arise from an individual’s effort to maintain synchronization with an auditory cue, requiring them to adjust their step dynamics to match a specific beat. Because step width is closely related to balance and stability, altering step width may be a compensatory mechanism that helps an individual maintain equilibrium whilst walking with cue-altered rhythm [21,22].

Although the precise mechanism underlying the variations in step width in this pilot study remains unclear, these preliminary findings help contribute to our understanding of the effects of different types of cueing on gait patterns and, subsequently, stability and fall risks. Increases in step width have been evidenced to coincide with reduced gait stability and control [22]; therefore, the reduction in step width under the music-cued condition of this study suggests greater stability. Previous studies have seen greater step widths in older populations compared to younger populations, as well as in individuals with higher incidence of falls [21]. These preliminary findings may have implications for the potential benefits of music-based interventions over metronome-cued or self-paced walking, and they may help tailor interventions to modulate gait parameters more effectively. Future research with a larger sample size and control population is needed to support this notion.

One key difference between this pilot study and those that have been conducted previously lies in the methodology of using self-paced walking speeds as opposed to speeds that matched metronome or music tempos. It was hypothesized that self-paced walking speeds could provide more optimal conditions for capturing natural gait patterns and observing the true effects of auditory cueing on various gait measures, as well as being more akin to the use of cueing in a clinical setting. A previous study, conducted by Luessi et al. [23], illustrated how walking faster than your preferred speed demands more attention and interferes with gait automaticity, an issue pronounced in PD. Thus, the use of cues may have a larger effect when gait speed is increased in persons with PD, possibly explaining the lack of effect at self-selected speeds revealed in this study. Studies examining differences in self-selected paces and faster walking paces are needed to parse out the most appropriate clinical application of using cues in PD.

Other differences between this pilot study and previous studies include sample size, statistical power, and characteristics of the specific population of PD participants recruited for this study. PD does not manifest in the same way from one individual to the next; severity and disease progression can vary drastically, so results of any study involving PD participants may be affected by varying symptomology. Previous studies, reviewed by Muthukrishnan et al. [6], highlighted how external cueing has different modes of action for individuals with varying PD severity. For example, small gait deviations seen in lower-severity PD can be offset with external cues, allowing individuals to maintain their normal gait pattern. However, higher-severity PD relies more heavily on external cues to improve gait and reduce risks of injury, but does not completely ameliorate pathologic gait symptoms [6]. In this pilot study, participants had mild to moderate PD, proving an additional possible explanation for the lack of effect of cueing revealed in this study.

### Limitations

This pilot study used a convenience sample which may have contributed to one of the main limitations of this study—small sample size. The small sample size and variability of the results are likely to have affected the ability to observe significant differences between gait measures, besides step width, with different cueing conditions. Continued recruitment and data collection was not feasible. Another limitation of this study is the absence of control group data. Changes related solely to PD cannot be fully understood from these results without comparison between matched healthy older adults and healthy younger adults to act as a control for the effects of aging. This limitation emphasizes the need for further research that includes matching healthy participants to better isolate the effects of auditory cueing on Parkinsonian gait. These challenges highlight areas for further research and provide a guide for the development of more refined PD gait studies and therapeutic interventions. Nonetheless, the main effect of condition on step width from this pilot study provides further preliminary evidence for understanding the use of auditory cues in gait in persons with PD. Additional factors, such as disease and music experience, were not explored to account for intra-group variability. As the music used was adjusted to the preferred walking pace, rhythm and familiarity with the music may have also been altered, potentially impacting motivation and the ability to synchronize with the music. However, no participant complained or expressed difficulty when walking to the adjusted music tempo. Finally, this study is limited to acute assessment, and long-term effects such as fall risk and quality of life that may have relevant clinical impact were not explored.

## 5. Conclusions

The preliminary findings of this pilot study contribute to a growing body of research and provide valuable insight into the effects of auditory cues on specific gait parameters. Although not supported by post-hoc significance, the observed reduction in step width highlights a potential effect of music on gait stability, which would have important implications for the design of therapeutic interventions for people with PD. Further research will allow us to deepen our understanding of how different types of auditory cues can impact gait and allow development of more optimal treatment strategies for peoples with PD to reduce risks of injury and improve overall quality of life. Such studies may include exploring individual variability to cues and how this correlates with clinical outcomes, evaluating the long-term impact of curing interventions, and standardizing the musical cues in regard to genre, style, rhythm, and familiarity. Taken together, a broader, controlled study with a personalized approach to take into account individual variability and sustainability of the effects remains necessary to define the real therapeutic potential of these interventions.

## Figures and Tables

**Table 1:** Participant demographics.

Participant	Sex	Age	Ethnicity	Handedness	MMSE	BDI	MDS UPDRS	PD Duration (Years)	Most -Affected Side	Medications	Music Experience (Years)	Type of Experience
1	F	73	White	Right	30	3	69	13	Left	C-L, Ropinirole	47	Piano
2	M	72	White	Right	30	6	70	12	Left	Azilect, Amantadine	13	Singing, guitar
3	F	63	White	Right	29	8	76	4	Left	C-L	50+	Singing
4	F	64	White	Left	30	5	45	4	Left	C-L	10	Singing, guitar
5	F	63	White	Right	30	2	11	5	Right	Pramipexole, C-L	0	None
6	F	68	White	Right	30	8	67	16	Left	Azilect, THP, Amantadine	1	Piano
7	M	70	White	Right	28	9	76	4	Right	C-L	30	Choir
8	F	74	White	Right	30	9	51	7	Left	C-L	0	None
9	F	65	White	Right	30	1	20	2	Right	C-L	2	No Answer

C-L = Carbidopa–Levodopa, THP = Trihexyphenidyl, MMSE = Mini Mental State Exam, BDI = Beck Depression Inventory.

## Data Availability

The original contributions presented in this study are included in the article/Supplementary Material. Further inquiries can be directed to the corresponding author.

## Supplementary Materials

The following supporting information can be downloaded at: https://www.mdpi.com/article/10.3390/brainsci15050528/s1, Table S1. Raw data: Spatiotemporal measures of gait.

## Abbreviations

The following abbreviations are used in this manuscript:

PD	Parkinson’s Disease
MMSE	Mini-Mental State Exam
BDI	Beck Depression Index
MDS-UPDRS	Movement Disorder Society–Unified Parkinson’s Disease Rating Scale
C-L	Carbidopa–Levodopa
THP	Trihexyphenidyl
SMA	Supplementary Motor Area

## References

[1] Constantinescu R., Leonard C., Deeley C., Kurlan R. (2007). Assistive devices for gait in Parkinson’s disease. Park. Relat. Disord..

[2] Chen P.-H., Wang R.-L., Liou D.-J., Shaw J.-S. (2013). Gait disorders in Parkinson’s disease: Assessment and management. Int. J. Gerontol..

[3] McNeely M.E., Duncan R.P., Earhart G.M. (2012). Medication improves balance and complex gait performance in Parkinson disease. Gait Posture.

[4] Poláková K., Růžička E., Jech R., Kemlink D., Rusz J., Miletínová E., Brožová H. (2020). 3D visual cueing shortens the double support phase of the gait cycle in patients with advanced Parkinson’s disease treated with DBS of the STN. PLoS ONE.

[5] Rocha P.A., Porfírio G.M., Ferraz H.B., Trevisani V.F.M. (2014). Effects of external cues on gait parameters of Parkinson’s disease patients: A schematic review. Clin. Neurol. Neurosurg..

[6] Muthukrishnan N., Abbas J.J., Shill H.A., Krishnamurthi N. (2019). Cueing paradigms to improve gait and posture in Parkinson’s Disease: A narrative review. Sensors.

[7] Schaefer R.S. (2014). Auditory rhythmic cueing in movement rehabilitation: Findings and possible mechanisms. Philos. Trans. R. Soc. B.

[8] Rose D., Delevoye-Turrell Y., Ott L., Annett L.E., Lovatt P.J. (2019). Music and metronomes differentially impact motor timing in people with and without Parkinson’s disease: Effects of slow, medium, and fast tempi on entrainment and synchronization performance in fingers tapping, toe tapping, and stepping on the spot tasks. Park. Dis..

[9] Wittwer J.E., Webster K.E., Hill K. (2013). Music and metronome cues produce different effects on gait spatiotemporal measures but not gait variability in healthy older adults. Gait Posture.

[10] Chawla G., Hoppe M., Browner N., Lewek M.D. (2021). Individuals with Parkinson’s disease retain spatiotemporal gait control with music and metronome cues. Mot. Control.

[11] Thaut M.H., McIntosh G.C., Rice R.R., Miller R.A., Rathbun J., Brault J.M. (1996). Rhythmic auditory stimulation in gait training for Parkinson’s disease patients. Mov. Disord..

[12] Ready E.A., Holmes J.D., Grahn J.A. (2022). Gait in younger and older adults during rhythmic auditory stimulation is influenced by groove, familiarity, beat perception, and synchronization demands. Hum. Mov. Sci..

[13] Morris M.E., Iansek R., Matyas T.A., Summers J.J. (1996). Stride length regulation in Parkinson’s disease: Normalization strategies and underlying mechanisms. Brain.

[14] Hoddinott J.D., Grahn J.A. (2024). Neural representations of beat and rhythm in motor and association regions. Cereb. Cortex.

[15] Rahimpour S., Rajkumar S., Hallett M. (2021). The supplementary motor complex in Parkinson’s Disease. J. Mov. Disord..

[16] Pesimena G., Wilson C.J., Bertamini M., Soranzo A. (2019). The role of perspective taking on attention: A review of the special issue on the reflexive attentional shift phenomenon. Vision.

[17] Ashoori A., Eagleman D.M., Jankovic J. (2015). Effects of auditory rhythm and music on gait disturbances in Parkinson’s Disease. Front. Neurol..

[18] Peterson D.S., Smulders K. (2015). Cues and attention in Parkinsonian gait: Potential mechanisms and future directions. Front. Neurol..

[19] Repp B.H., Su Y.H. (2013). Sensorimotor synchronization: A review of recent research (2006–2012). Psychon. Bull. Rev..

[20] Hove M.J., Suzuki K., Uchitomi H., Orimo S., Miyake Y. (2012). Interactive rhythmic auditory stimulation reinstates natural 1/f timing in gait of Parkinson’s patients. PLoS ONE.

[21] Alizadehsaravi L., Bruijn S.M., Muijres W., Koster R.A.J., van Dieën J.H. (2022). Improvement in gait stability in older adults after ten sessions of standing balance training. PLoS ONE.

[22] Molina L.K., Small G.H., Neptune R.R. (2023). The influence of step width on balance control and response strategies during perturbed walking in healthy young adults. J. Biomech..

[23] Luessi F., Mueller L.K., Breimhorst M., Vogt T. (2012). Influence of visual cues on gait in Parkinson’s disease during treadmill walking at multiple velocities. J. Neurol. Sci..

