# Supplementary material for: A Pilot Study on the Influence of Self-Paced Auditory Cues and Preferred Music on Gait in Persons with Parkinson’s Disease
Source: Brain Sci. 2025 May 20;15(5):528. doi: 10.3390/brainsci15050528 (PMC12110284; doi:10.3390/brainsci15050528)
Supplement: Supplementary file 1 [file brainsci-15-00528-s001.zip › brainsci-3565045-supplementary.pdf]

Supplementary Table S1. Raw Data: Spatiotemporal Measures of Gait

Participant	Cadence (steps/min)	Step Time (seconds)	Step Length (cm)	Step Width (cm)	Stride Length (cm)	Swing Time (seconds)	Stance Time (seconds)	Single Supp. Time (seconds)	Double Supp. Time (seconds)
**Self-Paced Walking Condition**									
1	105.7	0.566	64.3455	8.5095	129.0995	0.4545	0.6775	0.4545	0.2245
2	104.1	0.577	52.8725	6.875	105.772	0.436	0.7205	0.436	0.292
3	97.8	0.6135	63.813	11.3935	127.6735	0.455	0.7685	0.455	0.3145
4	114.2	0.526	52.633	6.4535	105.3365	0.396	0.655	0.396	0.263
5	128.6	0.467	80.413	10.284	161.1195	0.3975	0.536	0.3975	0.142
6	114.8	0.523	64.202	3.758	128.5495	0.422	0.621	0.422	0.1995
7	104.9	0.5755	60.386	14.688	120.9325	0.4385	0.718	0.4385	0.2855
8	93.9	0.639	48.531	21.136	97.8665	0.4815	0.8055	0.4815	0.331
9	103.2	0.58	64.791	7.3895	129.942	0.4675	0.691	0.4675	0.2175
**Cued Walking Condition**									
Participant	Cadence (steps/min)	Step Time (seconds)	Step Length (cm)	Step Width (cm)	Stride Length (cm)	Swing Time (seconds)	Stance Time (seconds)	Single Supp. Time (seconds)	Double Supp. Time (seconds)
1	106.2	0.5655	68.167	8.7125	136.615	0.4575	0.6755	0.4575	0.221
2	106.4	0.564	56.3375	7.6025	112.943	0.4295	0.6985	0.4295	0.273
3	78.2	0.767	62.084	9.22	125.0265	0.568	0.968	0.568	0.405
4	114.3	0.525	51.893	7.1045	103.8745	0.388	0.6615	0.388	0.2775
5	138.1	0.4345	72.4785	9.316	143.7585	0.358	0.509	0.358	0.153
6	115.5	0.518	66.9335	4.329	134.1075	0.4255	0.6095	0.4255	0.1905
7	109.1	0.549	63.803	13.8885	128.621	0.431	0.668	0.431	0.2435
8	93.9	0.64	51.967	18.9605	104.158	0.4715	0.8055	0.4715	0.3355
9	107.5	0.5565	66.2845	6.8235	133.5105	0.4635	0.6545	0.4635	0.2075
**Music Walking Condition**									
Participant	Cadence (steps/min)	Step Time (seconds)	Step Length (cm)	Step Width (cm)	Stride Length (cm)	Swing Time (seconds)	Stance Time (seconds)	Single Supp. Time (seconds)	Double Supp. Time (seconds)
1	104.9	0.5725	68.9185	7.185	137.972	0.4595	0.689	0.4595	0.228
2	107	0.5615	58.591	8.1745	117.5235	0.4325	0.6915	0.4325	0.264
3	78.9	0.76	59.9305	9.3535	119.9795	0.549	0.977	0.549	0.427
4	116.7	0.514	53.3705	7.272	106.7055	0.386	0.642	0.386	0.2615
5	133.2	0.451	73.06	8.7105	146.5315	0.379	0.522	0.379	0.1475
6	117.6	0.509	64.3885	4.083	129.178	0.41	0.608	0.41	0.192
7	105.1	0.571	61.3345	13.165	122.896	0.4405	0.702	0.4405	0.2605
8	94.9	0.6315	49.094	17.713	98.4135	0.463	0.8	0.463	0.341
9	105.3	0.5675	67.779	5.298	135.9505	0.4655	0.6655	0.4655	0.2025
